# Optimization of a Molecularly Imprinted Polymer Synthesis for a Rapid Detection of Caffeic Acid in Wine

**DOI:** 10.3390/foods12081660

**Published:** 2023-04-16

**Authors:** Marie Elhachem, Elias Bou-Maroun, Maher Abboud, Philippe Cayot, Richard G. Maroun

**Affiliations:** 1 PAM UMR A 02.102, Institut Agro, Université Bourgogne Franche-Comté, 1 Esplanade Erasme, F-21000 Dijon, France; 2 Centre d’Analyses et de Recherche, Laboratoire CTA, UR TVA, Faculty of Sciences, Saint Joseph University, Beirut 1104 2020, Lebanon; 3 UEGP Unité Environnement, Génomique et Protéomique, Faculty of Sciences, Saint Joseph University, BP 17-5208 Mar Mikhael, Beirut 1104 2020, Lebanon

**Keywords:** molecularly imprinted polymers, synthesis optimization, electrochemistry, wine, antioxidants, fast method, phenolic compounds, screen printed electrode

## Abstract

Molecular imprinting is an efficient strategy to make the detection of compounds more specific and more selective. This targeted analytical strategy using molecularly imprinted polymer (MIP) synthesis needs to obtain the optimized conditions. A selective molecularly imprinted polymer was prepared for caffeic acid (CA) detection after varying the following synthesis parameters: functional monomer type (N-phenylacrylamide, N-PAA or methacrylic acid, MAA), solvent type (acetonitrile/methanol or acetonitrile/toluene), and the polymerization method (UV or thermal initiation). The optimal polymer was obtained using MAA as a functional monomer, acetonitrile/methanol as solvent, and UV polymerization. Morphological characterizations were done for the optimal CA-MIP using mid-infrared spectroscopy, scanning electron microscopy, and nitrogen adsorption. The optimal polymer showed good specificity and selectivity in the presence of interferents (antioxidants having a chemical structure close to CA) in a hydroalcoholic solution. The electrochemical detection of CA was performed by cyclic voltammetry (CV) after the interaction between CA and the optimal MIP in a wine sample. The linear range of the developed method was between 0 and 1.11 mM, the limit of detection (LOD) was 0.13 mM, and the limit of quantification (LOQ) was 0.32 mM. HPLC-UV was used to validate the newly developed method. Recovery values were between 104% and 111%.

## 1. Introduction

The presence of antioxidants in foods, beverages, cosmetics, and pharmaceuticals has become of great importance to both producers and consumers. The latter is becoming increasingly aware of their health benefits [1,2,3]. Research in the field of antioxidants is more and more focused on the valorization of these compounds, their extraction, detection, identification, and monitoring. Liquid chromatography coupled to a UV or mass spectrometry detector is the most used classical detection method for antioxidants. They are, in general, time-consuming and need a sample preparation step to eliminate the interference [4,5,6,7,8,9].

Caffeic acid (CA) is an orthodiphenol that belongs to the subgroup of hydroxycinnamic acids, it was chosen for its antioxidant characteristics and because it is widespread in plants (rosemary), fruit (wild blueberries, blackcurrant), and drinks (ginko infusion, tea, wine, and coffee). It is a quality tracer of wine oxidation. Potatoes peels or fruit peels are rich in caffeic acid that can be valorized after an extraction step [10,11,12,13,14].

A rapid method has been presented by Elhachem et al. [15] for the electrochemical detection of caffeic acid using molecularly imprinted polymers (MIP) and screen-printed electrodes and has been applied in wine. This simple and rapid method showed interesting results in terms of selectivity and was validated by a reference method, the HPLC-UV. However, the developed method presented a lack of specificity, traduced by a slight difference between the imprinted and non-imprinted polymer. For this reason, it is important to optimize the synthesis of this polymer couple in order to keep its selectivity and improve its specificity.

The MIP synthesis is, in general, easy to perform but requires a long procedure for the optimization of its conditions and reagents. A lot of parameters can influence the synthesis, starting with the reagents type (template, functional monomer, cross-linker, initiator), the solvent nature (protic, aprotic), the radical initiation method (thermal and UV), the type of polymerization (precipitation, mass, bulk), the drying conditions, and the washing method for the template extraction [16,17,18]. Once the influencing parameters are set up, the rebinding tests are then performed to study the MIP performance.

In this work, eleven CA-MIP/NIP couples were synthesized by varying the following parameters: the functional monomer (MAA or N-PAA), the solvent (acetonitrile/methanol or acetonitrile/toluene), and the polymerization method (UV or thermal). Rebinding characteristics of all 11 couples of MIP and NIP were studied in the hydroalcoholic medium. The objective was to choose the optimal couple (having the highest specificity, i.e., the highest difference in terms of adsorption between MIP and NIP) and use it to detect caffeic acid in wine using a fast method based on cyclic voltammetry (CV). The optimal polymer was characterized by FTIR, scanning electron microscopy, and nitrogen sorption isotherms. Selectivity tests were performed using the optimal MIP in a hydroalcoholic solution and four interferent antioxidants having a close chemical structure to CA. The newly developed method (MIP coupled to CV) was applied in a Burgundy red wine in order to determine CA and compared to the reference method HPLC-UV.

## 2. Materials and Methods

### 2.1. Chemicals

Caffeic acid (≥98%), N-phenylacrylamide (N-PAA, 99%), methacrylic acid (MAA, 99%), ethylene glycol dimethacrylate (EGDMA, 98%), azobisisobutyronitrile (AIBN, 98%), 2,2-Dimethoxy-2-phenylacetophenone (DMPAP, 99%), p-coumaric acid (≥98%), gallic acid, trans-ferulic acid (≥99%), sinapic acid (≥98%), acetonitrile (ACN, ≥99%), methanol (≥99%), toluene, phosphate-buffered saline (PBS tablets), ethanol (EtOH, ≥99%), and acetic acid (100%) were purchased from Sigma Aldrich, France.

Water used in all experiments was deionized and obtained from an Elga Ionic system PURELAB Option, Vivendi Water, Antony, France. [15].

### 2.2. Instrumentation

A potentiostat µStat-i 400 (Meteohm, Villebon Courtaboeuf, France) was used for the cyclic voltammetry measurements. The analyses were performed with a scan rate of 50 mv/s, between −0.4 and +0.8 V. Screen-printed carbon electrodes SPCE DropSens 110 were purchased from Metrohm, Villebon Courtaboeuf, France. The anodic peak intensity Ipa was determined and used for quantification.

For the morphological characterization, Fourier transform infrared (FTIR) spectroscopy was recorded on a PerkinElmer spectrum 65 FT-IR spectrometer (PerkinElmer, Villebon-sur-Yvette, France) in the range 4000–500 cm^−1^ using attenuated total reflectance sampling. A total of 64 scans with a resolution of 4 cm^−1^ were applied.

Scanning Electron Microscopy (SEM) was applied for the surface morphological characterization using JEOL-7600 scanning electron microscope (Jeol Europe SAS, Croissy-sur-Seine, France).

Brunauer–Emmett–Teller (BET) method was used for surface area analysis and was performed by nitrogen adsorption-desorption at a temperature of 77 K using an ASAP 2020 instrument (Micromeritics, Merignac, France). Prior to analysis, 50 mg of the sample was outgassed for 14 h at room temperature [15].

### 2.3. Synthesis of Molecularly Imprinted and Non-Imprinted Polymers for Caffeic Acid (CA)

In order to obtain the optimal couple CA-MIP/NIP, eleven polymerizations were conducted by varying one of the following parameters: functional monomer type, solvent type, or the polymerization method. Table 1 shows the synthesis conditions, with one variation at a time.

Firstly, the template molecule (T: CA) was dissolved in the solvent using magnetic stirring. When methanol was used, CA was solubilized in it before adding acetonitrile. Then, the following reagents were added in the following order: functional monomer (FM: N-PAA or MAA), crosslinker (CL: EGDMA), and initiator. An interval of 5 min is respected between each addition of reagent to ensure their solubility. Nitrogen was used to degas the mixture for 20 min. A ratio of 1:4:20 was used for the T/FM/CL in both UV and thermal polymerizations.

In UV polymerization, 0.33 mmol of CA was added in a total volume of 33.3 mL of the solvents. The reaction mixtures were placed in tubes and fixed at 10 cm from a UV lamp (365 nm) for 24 h. In thermal polymerization, 0.5 mmol of CA was added in a total volume of 50 mL of the solvents. The polymerization occurred at 60 °C for 24 h. At the end of the polymerization, centrifugation (11,872× *g* for 10 min) was used to recover the polymer. The elimination of the template from the polymer to free the imprinted cavities was done using a washing solution composed of (ethanol/acetic acid, 90/10, *v*/*v*). The washing continued until no caffeic acid was detected by HPLC-UV in the washing solutions. The washed polymers were dried at 60 °C overnight and stored at room temperature before use.

### 2.4. Rebinding Experiments of Caffeic Acid in Hydroalcoholic Medium

Rebinding experiments were performed on both the MIP and NIP of all the couples. The quantity of adsorbed CA by the polymer was determined after varying the polymer mass from 10 to 50 mg. A certain mass of polymer was weighed in an Eppendorf tube. Afterward, 1 mL of PBS/ethanol (PBS 0.05 M pH 3/EtOH, 90/10, *v*/*v*) containing 1.11 mM of CA was added. The samples were placed on a rotator for 2 h (the stirring time was chosen based on a kinetic study where the rebinding properties of one sample were tested at various times of stirring, ranging from 5 min to 4 h) followed by centrifugation (10,640× *g* for 10 min).

For electrochemical measurements, 50 µL of the supernatant was dropped at the surface of a screen-printed carbon electrode, SPCE. The scan rate was 50 mV/s, the initial voltage was −0.4 V, and the final voltage was +0.8 V. The anodic peak current (Ipa) is proportional to the caffeic acid concentration remaining in the solution after the interaction with the polymer. The adsorbed caffeic acid (N_ads_, µmol) was calculated from the difference between the initial concentration of CA (C_0_, mM) and the remaining concentration (C, mM).

The concentration range of 0 to 2.78 mM of CA in PBS/EtOH was used to establish the calibration curve. Among all the synthesized MIP/NIP, couple number 6 showed the best performance and was chosen for the following applications.

### 2.5. Characterization of the CA-Polymer Using Adsorption Models

Langmuir isotherms represent one of the distribution models that represent the kinetics of ligand binding to receptors. The binding equilibrium between CA [I] and the binding sites [S] is represented by the following equation:

I + S → IS(1)

Equation (2) defines the binding affinity constant (K):

(2) $$K=\frac{\left[IS\right]}{\left[I\right][S]}$$

The relation between K and the number of binding sites (N_t_) is given in Equation (3):

(3) $$K=\frac{B}{F(N_{t}-B)}$$

(4) $$\frac{B}{F}=KN_{t}-KB$$

where B is the concentration of bound CA and F is the concentration of free CA at equilibrium. They are calculated based on the rebinding properties experiment applied in the model wine spiked with CA. The Scatchard plot B/F = f(B) (Equation (4)) allows the determination of K and N_t_ after linear regression and the determination of the slope and the intercept [19].

### 2.6. Selectivity Tests in Hydroalcoholic Medium

Selectivity tests were performed in order to test the response of the polymer towards CA and other interferents with similar chemical structures. A total of 10 mg of MIP6/NIP6 were added to 1 mL of PBS/EtOH containing 0.55 mM of each of the following compounds: caffeic acid, sinapic acid, ferulic acid, p-coumaric acid, and gallic acid. These antioxidants are commonly found in wine. The samples were then stirred and centrifuged as described in Section 2.4, the supernatants were deposited on the SPCE, and CV measurements were applied.

### 2.7. Rebinding Experiments of Caffeic Acid in Wine

The rebinding properties were tested again, but this time in wine samples. A Burgundy red wine (Pinot Noir, 2020) was purchased from a local supermarket.

The wine was diluted 10 times in PBS/EtOH and spiked with different concentrations of caffeic acid ranging between 0 and 1.11 mM. The rebinding properties were tested using the same previous procedure described in Section 2.4 by adding 1 mL of the spiked wine in different Eppendorf tubes in the presence of 10 mg of polymer. In parallel, another test was applied by fixing the CA concentration at 0.056 mM (it represents an average concentration of CA present in red wine [20]) and varying the polymer mass: 10; 20; 30; 50; 60; and 80 mg. The variation of the polymer mass helps to estimate the amount of polymer required for the rebinding of CA present in the medium. After stirring and centrifugation, the supernatants were deposited on the SPCE, and voltammetry measurements were applied.

The following concentration range was used to establish the calibration curve in diluted wine: 0 to 1.665 mM of CA. The equation of the calibration curve was y = 0.1988x + 2.5632, with a correlation coefficient R^2^ = 0.9989 (y represents Ipa in µA, and x represents the caffeic acid concentration in mM).

### 2.8. CA Determination in Wine Samples Using HPLC

HPLC was used as a conventional method to determine CA concentration in the same wine samples that were previously analyzed using voltammetry measurements. The HPLC alternative method was used to evaluate the efficiency of the voltammetry-based method. A Restek column (Pinacle II, C18 5 μm, 150 × 4.6 mm) and a Shimadzu LC (LC-20AT) equipped with a UV visible absorbance detector (SPD-20A) were used. The chromatographic separation was achieved with a gradient of two solvents: (solvent A 0.1% *v*/*v* acetic acid in water, solvent B 0.1% *v*/*v* acetic acid in acetonitrile) 7.5% of B from 0 to 8 min, 95% from 8 to 10 min, and 7.5% from 10 to 12 min. The flow rate was 1.0 mL.min^−1^, and detection of CA was performed at 325 nm [15].

## 3. Results and Discussion

### 3.1. Synthesis of Imprinted and Non-Imprinted Polymers

Two functional monomers were chosen for the synthesis. N-acrylamide was already used in a previous study (CA-MIP1/NIP1) [15]. Its chemical structure, having an aromatic ring and hydroxyl groups, allows it to form π–π stacking and hydrogen bonds with the template. Methacrylic acid is a well-known functional monomer commonly used in radical polymerization because of its high interaction with the acid template.

As preliminary results, the solubility of CA in the solvent, the presence or absence of the polymerization, and the resulting polymer yield were assessed before the rebinding experiments of the polymers. The results are shown in Table 2.

The solubility of the template in the solvent is essential because it is the starting point of the polymerization that promotes template-monomer interactions. Table 2 shows that when the template was not soluble, no polymerization of MIP was obtained (couples 4, 7, 8, and 9), or if it happened, it formed non-specific cavities that make MIP and NIP responses close to each other (couples 3, 10, and 11). The obtained yield is not the most important parameter, but a high yield is still valuable because it avoids the need to make a new synthesis for additional applications and reduces the synthesis cost. Finally, the most important parameter is the specificity of the polymer, which is represented in Table 2 by ΔN_ads_ and % of NIP adsorption in the presence of 10 mg of polymer. ΔN_ads_ is the difference between the amount of CA retained by MIP and the one retained by NIP. The increase of ΔN_ads_ indicates an increase in specificity. The percentage of NIP adsorption is defined by the amount of CA retained by the NIP. The lower this value, the higher the specificity. Couples five and six showed the best specificity with a low or no adsorption by the NIP6 in comparison with MIP6. Although both couples had MAA as a functional monomer. The type of radical initiation is an additional factor that helped determine the best couple. Couple six showed a better specificity due to UV polymerization compared to thermal initiation (couple five). UV polymerization contributes better to the formation of the cavities and the stability of the polymer.

### 3.2. Optimization of the CA-MIP/NIP Synthesis

**Figure 1:**
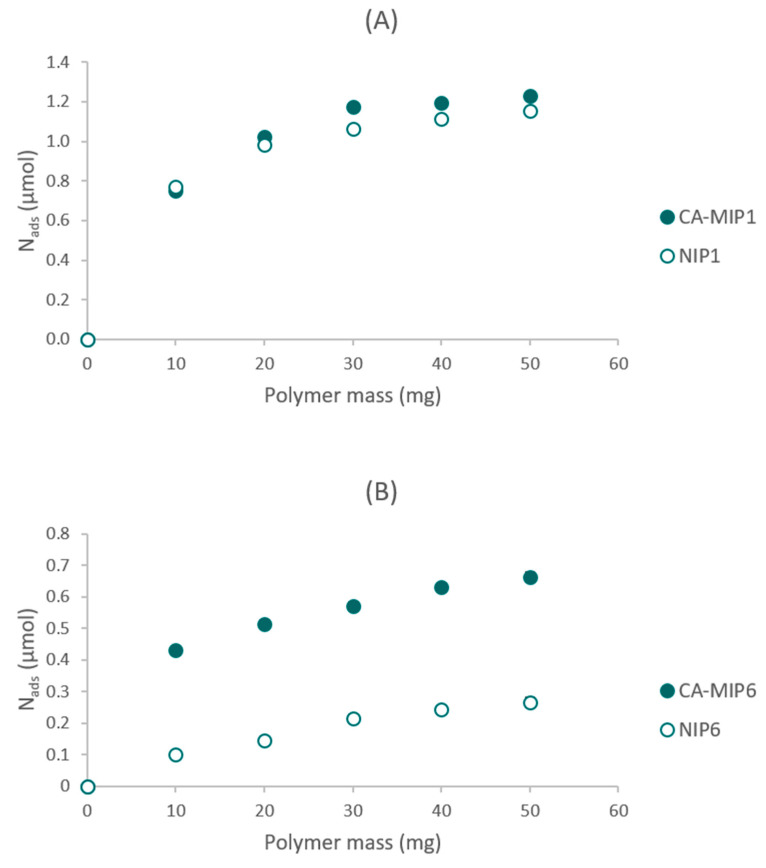
(**A**) Rebinding experiments of CA-MIP1/NIP1, (**B**) Rebinding experiments of CA-MIP6/NIP6.

Eleven couples of CA-MIP/NIP were synthesized. The optimal polymer was chosen based on its performance in the presence of a caffeic acid solution, and especially the difference between MIP and NIP. Compared to the MIP1/NIP1, the couple MIP6/NIP6 had the highest difference between the rebinding capacity of the MIP and NIP (Figure 1). N_ads_ represent the amount of caffeic acid adsorbed by the polymer.

The difference between the syntheses of MIP6/NIP6 and the other polymers remains in the functional monomer (FM) and the type of radical initiation. The results presented in Figure 1 show that MAA, used for the synthesis of couple six, improved the difference between the imprinted and non-imprinted polymers. MAA seems to be a better functional monomer than N-PAA due to the interaction between the acid function of MAA and the acid function of the template. The π–π stacking contributes to a lesser extent to the difference between MIP and NIP. In MIP/NIP6, a UV polymerization was used rather than a thermal polymerization. Thus, the difference between MIP6/NIP6 prepared at room temperature is better than that of the difference between MIP1/NIP1 obtained at 60 °C. The specific cavities, which are at the origin of the difference between MIP and NIP, seem to be more structured and better formed at low temperatures.

### 3.3. Morphological Characterization

The MIP6 and NIP6 were characterized by Fourier transform infrared (FTIR) spectroscopy, Scanning Electron Microscopy, and the BET method.

**Figure 2:**
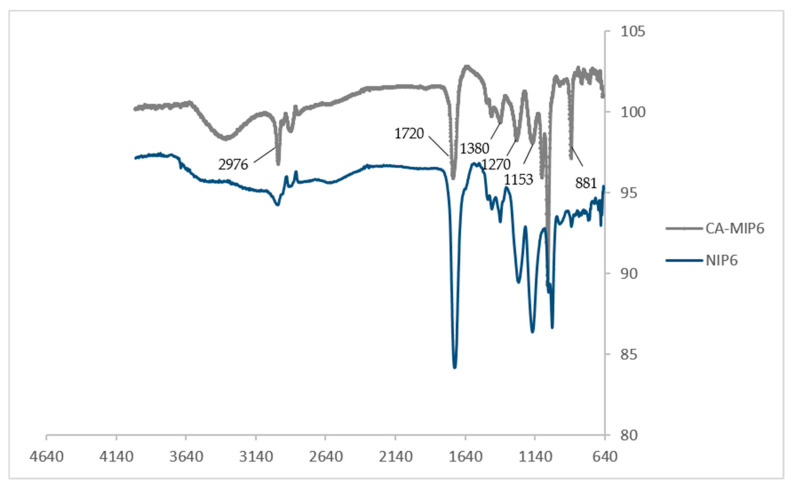
FTIR spectra of CA-MIP6 and NIP6.

Figure 2 showed that most of the obtained peaks come from the crosslinker EGDMA and the functional monomer MAA. The peak for MIP6 at 881 cm^−1^, attributed to the C-H out-of-plane bending from phenyl rings, can be due to the presence of the template (CA) during the synthesis. The main absorption bands were at 2976 cm^−1^ (C-H stretching), 1720 cm^−1^ (C=O stretching in EGDMA and MAA), 1056, 1085, 1153, 1270, and 1380 cm^−1^ (C-O stretching) [21]. The similarity of the two spectra MIP6 and NIP6 is explained by the fact that the major constituents of MIP6 and NIP6 are EGDMA and MAA.

**Figure 3:**
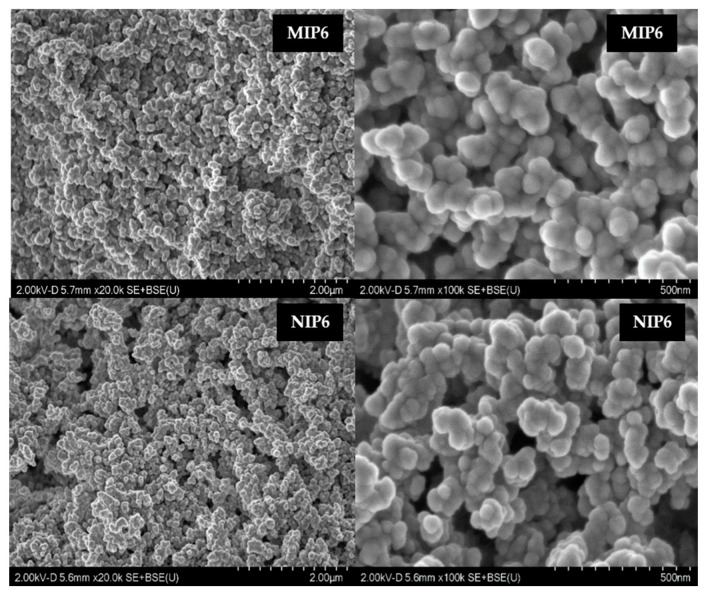
SEM images for MIP6 and NIP6 at different scales.

SEM micrographs (Figure 3) showed aggregated particles for MIP6 and NIP6. The particle size for these two polymers is around 10–20 nm. These values are 20 to 40 times less than the size of MIP1/NIP1.

The specific surface area of the MIP and the NIP was determined using the BET method. For MIP1, it was 9.9 m^2^.g^−1^, and for NIP1, it was 8.3 m^2^.g^−1^ [15]. For MIP6, it was 82.4 m^2^.g^−1^, and for NIP, it was 89.0 m^2^.g^−1^. The N_2_ sorption isotherms for MIP6 and NIP6 do not show any difference and are shown in the supplementary data (Figure S1). They are of type-III, indicating that they are powders with pore diameters exceeding micropores. The conditions of the optimal couple MIP/NIP have clearly increased the specific surface area of the sample. This could be due to the better formation of the specific cavities in MIP6 in comparison to MIP1.

### 3.4. Analysis of Binding Properties: Langmuir Isotherm

**Figure 4:**
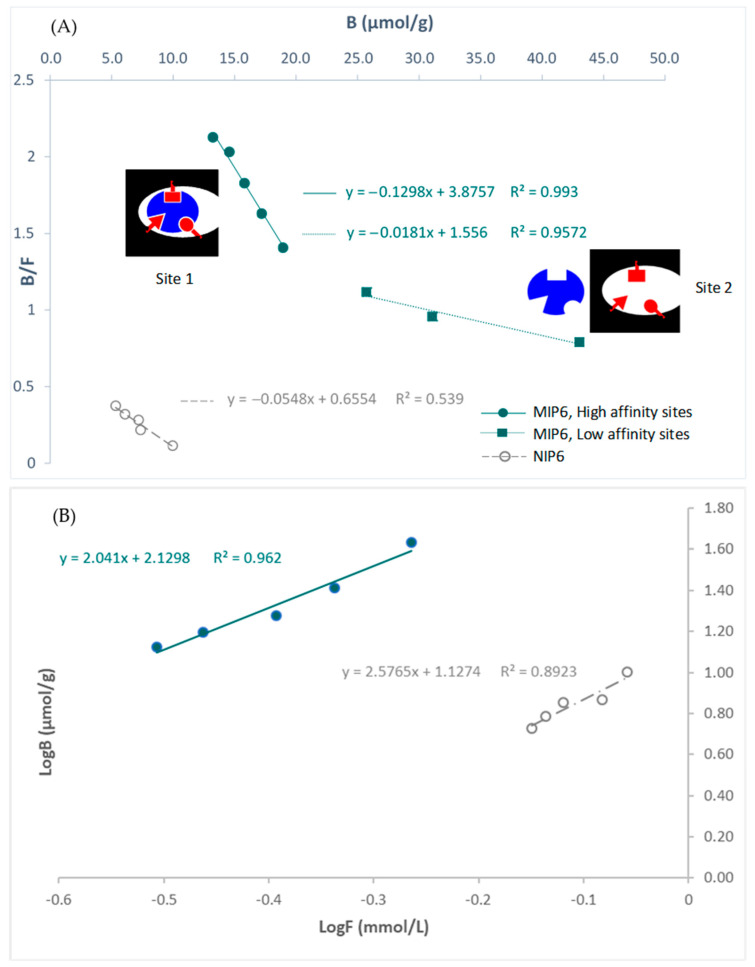
(**A**) Scatchard plots for MIP6 and NIP6, and (**B**) affinity distributions plotted in log format. Site 1: high-affinity site. Site 2: low-affinity site.

The Scatchard plot showed that the recognition sites of the imprinted polymer are heterogeneous: (site 1: high-affinity sites) and (site 2: low-affinity sites) (Figure 4). However, the non-imprinted polymer showed homogeneous sites.

The slope of the Scatchard plots of MIP-6 allowed the determination of K1: binding constant of the high-affinity sites (K1 = 0.1298 µmol/L). It was about seven times greater than K2: binding constant of the low-affinity sites.

Site 1 could be due to the interaction of CA with the MIP inside the cavities, and site 2 to the interaction of CA with the MIP outside the cavities.

NIP6 presents only low-affinity sites. This result could explain the highest performance of MIP6 compared to NIP6 in terms of binding capacity. The intercept of the Scatchard plots of MIP-6 allowed the determination of N1: maximum number of interaction sites (N1 = 29.8 µmol.g^−1^).

### 3.5. The Behavior of the Optimal Polymer towards Other Interferents

**Figure 5:**
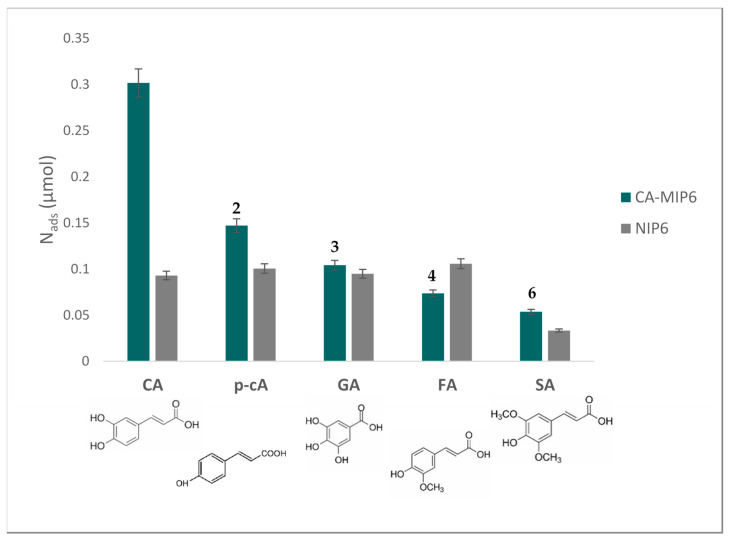
N_ads_ (mol number adsorbed on MIP6 and NIP6. Rebinding tests were done in a PBS/EtOH solution (PBS/EtOH, 90/10, *v*/*v*). Concentration of competing antioxidants = 0.55 mM, polymer mass = 10 mg. CA: caffeic acid, p-cA: p-coumaric acid, GA: gallic acid, FA: ferulic acid, SA: sinapic acid. The number above the MIP bars represents the selectivity coefficient α.

Cyclic voltammetry was used to assess the selectivity of MIP6 towards caffeic acid. In this experiment, the rebinding of five interferent antioxidants was assessed using MIP6 and NIP6. The results are presented in Figure 5.

Equation (5) provides the formula for the selectivity coefficient α:

(5) $$\alpha =\frac{N_{\mathrm{ads}}\;\mathrm{CA}}{N_{\mathrm{ads}}\;\mathrm{competing}\;\mathrm{antioxidant}}$$

α was calculated for each of the competing antioxidants. N_ads_ CA and N_ads_ competing antioxidants are the amounts of adsorbed CA and each of the competing antioxidants by MIP6, respectively.

Caffeic acid was the antioxidant that presented the highest adsorption on MIP6 compared with the other tested antioxidants. The selectivity coefficients varied from two for p-coumaric acid to six for sinapic acid. We believe that the orthodiphenol function of CA and its complementary interaction with the acid function of MAA and the size of CA are behind the selectivity. We also notice that the difference in the CA adsorbed amount between MIP6 and NIP6 is maintained. This difference was the highest among all the other antioxidants, which means that the imprinting process was successful.

### 3.6. Application in Wine, Recovery Tests, and Limit of Detection

**Figure 6:**
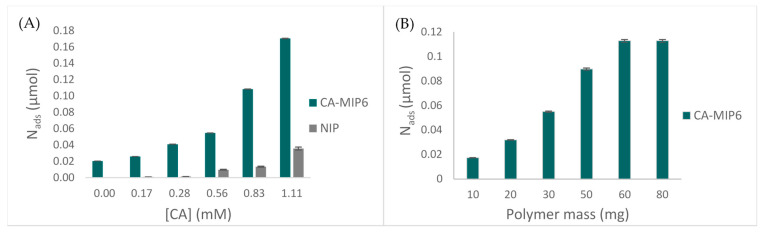
Rebinding properties of MIP6 in spiked wine: (**A**) N_ads_ of CA (µmol) in the function of the initial concentration, m of the polymer = 10 mg. (**B**) N_ads_ of CA (µmol) in the function of the polymer mass, initial CA concentration = 0.056 mM.

MIP6 was applied in a Burgundy red wine, in order to evaluate the binding properties towards caffeic acid. Figure 6A represents the variation of CA concentration for 10 mg of polymer. The results show an increase of rebinding with an important difference between MIP6 and NIP6. No difference between MIP and NIP is observed for concentrations below 0.56 mM, which is the maximum CA concentration found in wine. Meanwhile, Figure 6B shows that 60 mg of the polymer was necessary to reach the maximum adsorption.

The same samples prepared with different concentrations of CA were injected in HPLC-UV in order to validate the efficiency of our method. Equation (6) provides the recovery percentage by comparing the concentration of CA determined by CV to the one determined by HPLC.

(6) $$\mathrm{Recovery}\;(\%)=\frac{{\left[CA\right]}_{found\;in\;CV}}{{\left[CA\right]}_{found\;in\;HPLC}}\times 100$$

Table 3 presents the comparison between the newly developed method (MIP6/cyclic voltammetry) and the reference method (HPLC-UV).

The recovery values ranged between 104% and 111%. They are in line with a similar study performed by Figueirêdo Leite et al. on the determination of caffeic acid in wine using an electrochemical sensor based on a molecularly imprinted siloxanes [22]. This indicates that the CV method is accurate and may be promising for future commercial applications. Recovery values higher than 100% could be explained by the fact that CA-MIP6, although it interacts mainly with caffeic acid, is able to adsorb structurally related interferents. However, the values for percentage recovery remain acceptable for analytical applications.

Equations (7) and (8) explain how the limit of detection (LOD) and the limit of quantification (LOQ) were calculated for the MIP6/cyclic voltammetry method [23].

(7) $$\mathrm{LOD}=\frac{\left(\mathrm{intercept}+3s\right)}{\mathrm{slope}}$$

(8) $$\mathrm{LOQ}=\frac{\left(\mathrm{intercept}+10s\right)}{\mathrm{slope}}$$

The intercept was determined based on the calibration curve in wine, and “s” is the standard deviation corresponding to the intercept.

The calculated LOD for the MIP6/cyclic voltammetry method was 0.13 mM, and the calculated LOQ was 0.32 mM. These values are low enough to determine caffeic acid in wine samples.

## 4. Conclusions

In this work, several synthesis parameters were varied to obtain a molecularly imprinted polymer specific to caffeic acid. For this reason, 22 imprinted and non-imprinted polymers were synthesized by varying the functional monomer type, the solvent nature, and the polymerization method. CA was determined by a fast method using a screen-printed carbon electrode and cyclic voltammetry. The best polymer (optimal polymer, MIP6) was obtained using MAA as a functional monomer, acetonitrile/methanol as solvent, and UV polymerization. MAA played a key role because of the complementarity of its acid function with the acid function of caffeic acid. The UV polymerization at low temperatures was also important and was responsible for the success of the imprinting process. The Schatchard plot, which was realized on MIP6, showed the presence of two populations of binding sites inside the MIP: one of high affinity due to the interaction of CA with the MIP inside the imprinting cavities (interaction between CA and MAA, specific interaction) and one of low affinity due to the interaction of CA with the MIP outside the cavities (interaction between CA and the crosslinker; non-specific interactions). The selectivity of MIP6 was studied in a hydroalcoholic solution using four antioxidants with a close chemical structure to CA. The selectivity factor varied from two to six, showing that CA was the antioxidant with the highest affinity towards MIP6.

The optimal polymer was successfully applied in wine, and its performance was validated by HPLC-UV. The recovery range of the newly developed method varied between 104% and 111%, the LOD was 0.13 mM, and the LOQ was 0.32 mM. This new method is promising in terms of simplicity, efficiency, and rapidity compared to the conventional one.

In order to transform this method into a real-time tool for the determination of caffeic acid in an aqueous medium, an electropolymerization method where the functional monomer will be deposited directly on the surface of the electrode could be developed.

## Figures and Tables

**Table 1:** Condition synthesis of 11 couples of CA-MIP/NIP.

CA-MIP/NIP	T	FM	CL	I	S (80/20)	Type of Radical Initiation
1	CA	N-PAA	EGDMA	AIBN	ACN/MeOH	Thermal
2	CA	N-PAA	EGDMA	DMPAP	ACN/MeOH	UV
3	CA	N-PAA	EGDMA	DMPAP	ACN/Toluene	UV
4	CA	N-PAA	EGDMA	AIBN	ACN/Toluene	Thermal
5	CA	MAA	EGDMA	AIBN	ACN/MeOH	Thermal
6	CA	MAA	EGDMA	DMPAP	ACN/MeOH	UV
7	CA	MAA	EGDMA	AIBN	ACN/Toluene	Thermal
8	CA	MAA	EGDMA	DMPAP	ACN/Toluene	UV
9	CA	N-PAA	EGDMA	AIBN	ACN	Thermal
10	CA	N-PAA	EGDMA	AIBN	Toluene	Thermal
11	CA	MAA	EGDMA	AIBN	Toluene	Thermal

MIP: molecularly imprinted polymer, NIP: non-imprinted polymer, T: template, FM: functional monomer, CL: crosslinker, I: initiator, S: solvent, CA: caffeic acid, N-PAA: N-phenylacrylamide, MAA: methacrylic acid, EGDMA: ethylene glycol dimethacrylate, AIBN: azobisisobutyronitrile, DMPAP: 2,2-dimethoxy-2-phenylacetophenone, ACN: acetonitrile. UV at 365 nm for 24 h, thermal at 60 °C for 24 h.

**Table 2:** Resulting polymers characteristics before and after polymerization.

CA-MIP/NIP	Solubility of CA	Polymerization	Yield	ΔN_ads_ (µmol)	% of NIP Adsorption
1	YES	YES	CA-MIP1 43% NIP1 76%	0.04	94.2
2	YES	YES	CA-MIP2 12% NIP2 59%	0.06	94.3
3	NO	YES	CA-MIP3 33% NIP3 72%	0.02	97.9
4	NO	CA-MIP: NO NIP: YES	NIP4 80%	n.d.	n.d.
5	YES	YES	CA-MIP5 70% NIP5 93%	0.2	20
6	YES	YES	CA-MIP6 73% NIP6 88%	0.27	0
7	NO	NO	n.d.	n.d.	n.d.
8	NO	NO	n.d.	n.d.	n.d.
9	NO	CA-MIP: NO NIP: YES	NIP9 59%	n.d.	n.d.
10	NO	YES	CA-MIP10 53% NIP10 67%	0.08	92.2
11	NO	YES	CA-MIP11 87% NIP11 90%	−0.01	109

ΔNads (µmol) represents the difference between the adsorbed amounts of CA by MIP and NIP in the presence of 10 mg of polymer, and % of NIP adsorption represents the amount of caffeic acid retained by the NIP in the presence of 10 mg of polymer. n.d. = not determined.

**Table 3:** Percentage of CA determined by cyclic voltammetry using MIP6 and by HPLC-UV in comparison with the spiked quantity. The recovery percentage indicates how close these two values are.

Polymer Mass (mg)	Found [CA] in CV (%)	Found [CA] in HPLC-UV (%)	Recovery (%)
10	23	22	104
20	35	32	109
30	41	37	111

## Data Availability

The data are available from the corresponding author.

## Supplementary Materials

The following supporting information can be downloaded at: https://www.mdpi.com/article/10.3390/foods12081660/s1, Figure S1: N2 sorption isotherms of MIP6 and NIP6.
