# Supplementary figures and images for: Optimization of a Molecularly Imprinted Polymer Synthesis for a Rapid Detection of Caffeic Acid in Wine
Source: Foods. 2023 Apr 16;12(8):1660. doi: 10.3390/foods12081660 (PMC10137471; doi:10.3390/foods12081660)

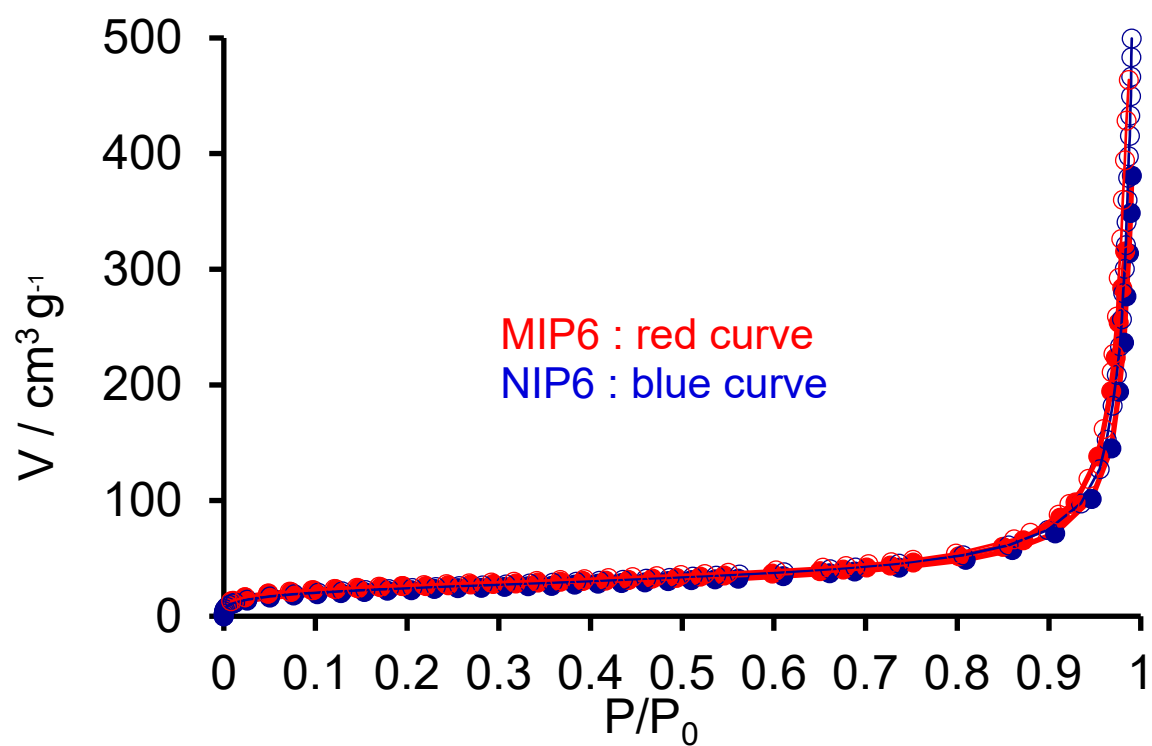

Figure S1. N<sub>2</sub> sorption isotherms of MIP6 and NIP6.

Supplement: Supplementary file 1 [file foods-12-01660-s001.zip › foods-2344212-supplementary.pdf]
